# Increased prevalence of Barrett’s esophagus in patients with *MUTYH*-associated polyposis (MAP)

**DOI:** 10.1007/s10689-020-00162-9

**Published:** 2020-02-22

**Authors:** Ceranza G. Daans, Zeinab Ghorbanoghli, Mary E. Velthuizen, Hans F. A. Vasen, George J. A. Offerhaus, Miangela M. Lacle, Peter D. Siersema, Margreet G. E. M. Ausems, Jurjen J. Boonstra

**Affiliations:** 1 Department of Infectious Diseases, Public Health Service of Amsterdam, Amsterdam, The Netherlands; 2 Department of Internal Medicine, Amsterdam UMC, VU University Medical Center, Amsterdam, The Netherlands; 3 Center of Expertise on Gender Dysphoria, Amsterdam UMC, VU University Medical Center, Amsterdam, The Netherlands; 4 The Netherlands Foundation for the Detection of Hereditary Tumours, Leiden, The Netherlands; 5 Department of Gastroenterology & Hepatology, Leiden University Medical Center, PO BOX 9600, 2300 RC Leiden, The Netherlands; 6 Department of Genetics, University Medical Center Utrecht (Location WKZ), Lundlaan 6, 3584 EA Utrecht, The Netherlands; 7 Department of Pathology, University Medical Center Utrecht, PO BOX 855000, 3508 GA Utrecht, The Netherlands; 8 Department of Gastroenterology, Radboud University Medical Center, PO BOX 9101, 6500 HB Nijmegen, The Netherlands

**Keywords:** Barrett’s esophagus, *MUTYH*-associated polyposis, Familial adenomatous polyposis, Esophageal adenocarcinoma

## Abstract

Barrett’s oesophagus (BE) has been associated with an increased risk of both colorectal adenomas and colorectal cancer. A recent investigation reported a high frequency of BE in patients with adenomatous polyposis coli (*APC*)-associated polyposis (FAP). The aim of the present study is to evaluate the prevalence of BE in a large cohort of patients with *MUTYH*-associated polyposis (MAP) and *APC*-associated adenomatous polyposis. Patients with a genetically confirmed diagnosis of familial adenomatous polyposis (FAP) or MAP were selected and upper gastrointestinal (GI) endoscopy reports, pathology reports of upper GI biopsies were reviewed to determine the prevalence of BE in these patients. Histologically confirmed BE was found in 7 (9.7%) of 72 patients with MAP. The mean age of diagnosis was 60.2 years (range 54.1–72.4 years). Two patients initially diagnosed with low grade dysplasia showed fast progression into high grade dysplasia and esophageal cancer, respectively. Only 4 (1.4%) of 365 patients with FAP were found to have pathologically confirmed BE. The prevalence of BE in patients with MAP is much higher than reported in the general population. We recommend that upper GI surveillance of patients with MAP should not only focus on the detection of gastric and duodenal adenomas but also on the presence of BE.

## Introduction

The incidence of esophageal adenocarcinoma (EAC) in Western populations has substantially increased over the past several decades. The majority of EACs is thought to derive from a precursor lesion—Barrett esophagus (BE). BE is characterized by the presence of columnar epithelium that has replaced the normal squamous cell lining of the distal esophagus. EAC develops through multistep progression from metaplasia into low grade dysplasia, high grade dysplasia, early adenocarcinoma, and, finally, invasive cancer. This metaplastic change is driven by chronic inflammation due to gastro-esophageal reflux disease (GERD), which is aggravated by abdominal obesity and smoking [1, 2]. In addition to environmental factors associated with BE and EAC, also genetic factors are thought to play a role [3, 4].

The prevalence of BE in asymptomatic patients varies between 0.5 and 1.8% and in patients with reflux symptoms, between 1.5 and 12.3% ([5–9], Table 1). It has been reported that BE and EAC are associated with a higher incidence of (sporadic) colorectal adenomatous polyps [10]. Also, familial adenomatous polyposis (FAP) caused by germline mutations in the adenomatous polyposis coli (*APC*) gene, has been associated with an increased risk of developing BE [11, 12]. It is not known whether adenomatous polyposis caused by bi-allelic germline mutations in the *MUTYH* gene (*MUTYH*-associated polyposis (MAP)) is also associated with BE [13]. The *MUTYH* gene plays an important role in base excision repair. Base excision repair is a cellular mechanism that repairs damaged DNA throughout the cell cycle. It is responsible primarily for removing small, non-helix-distorting base lesions from the genome [14]. EAC has been reported as part of the extracolonic tumor spectrum of MAP [15].

**Table 1:** The prevalence of Barrett’s esophagus (BE) reported in the general population (GP) and patients with and without gastro-esophageal reflux disease (GERD) symptoms

Study	Year	Country	Total number of patients	Prevalence of BE in GP (%)	Prevalence of BE in patients with GERD (%)	Prevalence of BE in patients without GERD (%)
Ronkainen et al.	2005	Sweden	1000	1.6	2.3	1.2
Zagari et al.	2008	Italy	1033	1.3	1.5	1.0
Peng et al.	2009	China	2580	1.0	–	0.5
Lee et al.	2010	South Korea	2048	1.0	12.3	0.5
Zou et al.	2011	China	1030	1.8	2.1	1.8

Surveillance of the upper gastrointestinal (GI)-tract is recommended for patients with MAP and FAP because of the increased risk of gastric and duodenal adenomas [16]. In the present study we assessed the prevalence of BE and EAC by reviewing the endoscopy reports in a large cohort of patients with FAP and MAP.

## Methods

Initially, the database of the Department of Genetics at the University Medical Centre Utrecht was used to identify patients diagnosed with a polyposis syndrome between November 1987 and April 2015. Patients with a genetically confirmed diagnosis of FAP or MAP were eligible for this study if one or more upper GI endoscopy reports and/or pathology reports of upper GI biopsies were available.

To increase the number of FAP and MAP patients, we also used data from the Dutch Hereditary Cancer Registry. This national registry, established in 1985, collect medical and pathology reports and reports of upper and lower GI endoscopy of all registered patients with FAP and MAP.

All available original upper-GI endoscopy reports were reviewed. Data on the presence of BE, length of BE and, if available, the Prague criteria (endoscopic grading system for BE) were recorded. Also, the presence of a hiatal hernia and GERD (based on the Los Angeles, LA classification [17]) were obtained. Secondly, the pathology reports of all included patients were collected from the PALGA (Dutch acronym for “Pathologisch-Anatomisch Landelijk Geautomatiseerd Archief”) database to confirm the histological diagnosis of BE. The PALGA database is a national automated archive where all pathology reports of all performed biopsies in the Netherlands are registered. BE was defined as esophageal columnar epithelium in the presence of goblet cells [18]. The available section slides of the Barrett biopsies were reviewed by an expert GI pathologist (GJHO and MML).

Only patients from the Department of Clinical Genetics that have given their informed consent for their medical records to be reviewed were included. All patients registered at the Dutch Hereditary Cancer Registry have given written informed consent for registration and use of their anonymous data for research.

Descriptive statistical analysis was used. Frequencies are presented as absolute numbers and percentages. Continuous data are presented as mean [standard deviation (SD)], and in the case of non-normally distributed data as median (range). Last follow-up was calculated as death, diagnosis of BE or end of the study.

## Results

### Prevalence of BE in patients with MAP

A total of 94 patients with genetically proven MAP were selected. In 72 of the 94 MAP patients, the upper GI endoscopy reports and/or pathology reports of upper GI biopsies were available, including 28 females and 44 males. The mean age at last follow-up was 60.9 years (range 27.3–87.6, SD 11.4) and the mean length of follow-up (in 60 of 72 MAP patients where data was available) was 10.1 years (range 0–26.2). Patients characteristics and endoscopic findings are shown in Table 2.

**Table 2:** Frequency of endoscopic findings in the esophagus in MAP and FAP-patients

	MAP patients (%)	FAP patients (%)
Total number of patients	72	356
Gender		
Female	28 (38.9)	179 (50.3)
Male	44 (61.1)	177 (49.7)
Age at last follow-up (range)	60.9 (27.3–87.6)	48.9 (30.3–86.0)
Endoscopic findings esophagus		
Histologically proven Barrett’s mucosa	7 (9.7)	4 (1.4%)
Esophagus adenocarcinoma	1 (1.4)	0
Other findings		
Gastro-esophageal reflux esophagitis	18 (25)	NA
Hiatal hernia	10 (14)	NA

*MAP MUTYH*-associated polyposis, *FAP APC*-associated polyposis. *NA* not available

A total of nine patients had an endoscopical diagnosis of BE, and in seven out of the nine patients, BE was confirmed by histology (Table 3). Revision of the section slides by an expert pathologist was possible in six out of seven patients, and in all six patients the diagnoses of BE was confirmed. Thus, the prevalence of pathologically confirmed BE in the total cohort was 9.7% (7/72). The seven patients with BE included five males and two females. The mean age at diagnosis of BE was 60.2 years (range 54.1–72.4 years, SD 6.5). The characteristics of the seven patients with BE are summarized in Table 3.

**Table 3:** Clinical, genetic and pathological characteristics of seven MAP patients with Barrett’s esophagus

Patient	Sex	Age at diagnosis (years)	Mutation 1	Mutation 2	Initial PA report	Revision
1	F	61	c.536A>G p.(Tyr179Cys)	c.638C>T p.(Pro213Leu)	No dysplasia	No dysplasia
2	M	72	c.1147delC, p.(Glu369Argfs*39)	c.1214C>T p.(Pro405Leu)	High grade dysplasia	High grade dysplasia
3	F	54	c.1187G>A p.(Gly396Asp)	c.1214C>T p.(Pro405Leu)	Low grade dysplasia	No dysplasia
4	M	58	c.536A>G p.(Tyr179Cys)	c.536A>G p.(Tyr179Cys)	Low grade dysplasia	Not available
5	M	57	c.536A>G p.(Tyr179Cys)	c.1214C>T p.(Pro405Leu)	No dysplasia	Indefinite for dysplasia
6	M	55	c.536A>G p.(Tyr179Cys)	c.933 + 3A >C splice site intron 10	No dysplasia	Indefinite for dysplasia
7	M	65	c.536A>G p.(Tyr179Cys)	c.1187G>A p.(Gly396Asp)	No dysplasia	No dysplasia

Information on previous endoscopies was available in six out of the seven patients with BE. In three patients, BE was diagnosed at the first upper-GI endoscopy. In the remaining three patients, the previous endoscopy, performed 1–4 years earlier, did not demonstrate evidence for BE.

**Fig. 1:**
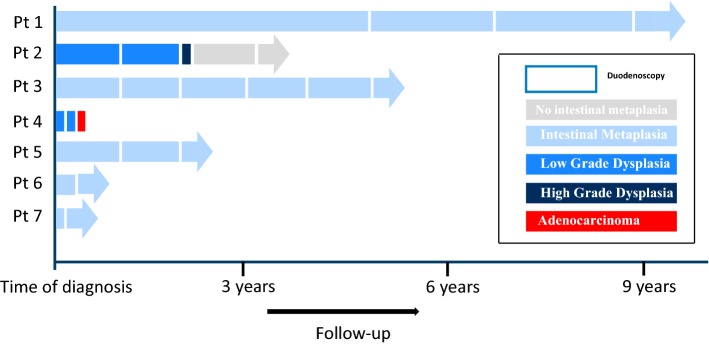
Findings at follow-up upper GI-endoscopies in the seven patients with Barrett’s esophagus. Arrow blocks represent screening intervals and the colors indicate the stage of metaplasia/dysplasia. *pt* patient. (Color figure online)

At time of diagnosis, two patients had low-grade dysplasia. The first patient developed high grade dysplasia after having low grade dysplasia in previous biopsies. The treatment consisted of piecemeal endoscopic mucosal resection. The second patient, initially diagnosed with low grade dysplasia, developed adenocarcinoma after 6 months and underwent a surgical resection of a pT2N2Mx EAC. The follow-up of the patients with BE are shown in Fig. 1.

### Prevalence of BE in patients with (A)FAP

In total, 407 FAP patients were identified from the 2 datasets. Upper GI endoscopy reports and/or pathology reports of upper GI biopsies were available in 356 patients including 177 males and 179 females. The mean age at the last follow-up was 48.9 years (range 30.3–86.0, SD 11.8).

In the total cohort, five patients with BE were detected. In four of these patients including two males and two females the diagnosis was confirmed by histological examination. In the fifth patient the diagnosis could not be confirmed as no goblet cells were present in the biopsies. The prevalence of histologically proven BE in this cohort is, therefore, 1.4%. The mean age at diagnosis of the four patients with BE was 52.5 years (range 34.0–60.0, SD 12.4). The endoscopic findings are summarized in Table 2.

## Discussion

The present study demonstrated a prevalence of BE (9.7%) in MAP patients which is > 5 times higher than reported in the general population. In contrast with a previous study, no increased frequency of BE was found in a large series of FAP-patients.

The prevalence of BE depends on which population is screened. In asymptomatic patients that undergo an upper GI endoscopy the prevalence varies between 0.5 and 1.8% and in patients with reflux symptoms, it is between 1.5 and 12.3% (Table 1; [5–9]). The proportion of MAP patients in our series with gastro-esophageal reflux esophagitis (25%) is not higher than reported in the general population which suggests that the frequency of BE is not increased by selection of symptomatic patients.

Another interesting finding was that in this small cohort of BE patients, two of the seven patients with initial low-grade dysplasia showed fast progression to high grade dysplasia and EAC, respectively. From a biological point of view our findings seem plausible. Persistent inflammation in esophageal mucosa due to acids and bile acids is associated with DNA impairment caused by increased formation of reactive oxygen species (ROS) [19–21]. One of the main defensive mechanisms to eliminate ROS induced DNA damage in cells is base-excision repair. Since *MUTYH* protein is a key player in base-excision repair, loss of the *MUTYH*-proteins could lead to accumulation of mutations and finally drive oncogenesis.

Analysis of our cohort of 356 FAP patients revealed that the prevalence of BE (1.4%) is not higher than in the general population. This is in contrast with a previous report on 36 (A)FAP patients of whom 6 (16%) had histologically proven BE [11]. We do not have an explanation for the observed differences but in view of the relatively small number of patients in the previous report, the findings might be due to chance. The fact that EAC has only been reported as part of the tumor spectrum of MAP but not in FAP supports our findings.

The strength of this study is the large number of patients with MAP and FAP and the long follow-up time.

In addition, all pathology reports were cross linked with the National Database (PALGA) and all biopsies of patients with BE were reviewed by an expert pathologist. There are also some limitations. At first, it is a retrospective analysis which might have led to selection of patients with BE. Secondly, not all risk-factors for the development of BE could be collected, such as smoking, obesity, symptoms of GERD or alcohol use.

What is the clinical implication of our study? Based on our observations, we recommend that upper GI surveillance of patients with MAP should not only focus on the identification of gastric and duodenal adenomas but also on the presence of BE. In view of the observed acceleration of high-grade dysplasia and EAC development, more intensive follow-up might be considered in patients with BE. In conclusion, this study demonstrates that the prevalence of BE with patients with MAP is much higher compared to the general population. This can be explained by the impaired *MUTYH* protein function that plays a role in the repair of DNA damage caused by oxidative stress such as GERD.
